# The “Silent” Threat: Group G Streptococcus Peritonitis in Peritoneal Dialysis

**DOI:** 10.7759/cureus.73479

**Published:** 2024-11-11

**Authors:** Lucinda Lau, Stefano Benincasa, Rachel Nash

**Affiliations:** 1 Internal Medicine, Cooper Medical School of Rowan University, Camden, USA; 2 Hospital Medicine, Cooper University Hospital, Camden, USA

**Keywords:** case report, ggs bacteremia, group g streptococcus (ggs), peritoneal dialysis related peritonitis, silent peritonitis

## Abstract

Peritoneal dialysis (PD) is a therapy for patients with end-stage renal disease (ESRD). PD carries an increased risk of peritonitis, often secondary to poor technique. A small subset of patients may present with “silent” peritonitis, or peritonitis in the absence of fever and abdominal pain, making diagnosis and treatment challenging. Identification of a causative organism can be an added barrier further delaying care. We present the case of a 95-year-old female with a history of ESRD on PD who presented with fatigue, confusion, and a lower leg wound found to have group G Streptococcus bacteremia. Treatment with intravenous antibiotics did not yield significant improvement, prompting the investigation of PD dialysate as a potential source. The patient was afebrile with persistent leukocytosis and no clinical signs of peritonitis. Yet, peritoneal fluid analysis confirmed peritonitis. This case underscores the importance of remaining cognizant of PD peritonitis in patients receiving PD therapy even in the absence of classic symptomatology. Risk factors such as advanced age, dementia, altered mentation from sepsis, or uncommon organisms may create an atypical presentation, delaying diagnosis and treatment. High rates of morbidity in peritonitis with concomitant bacteremia make early treatment even more crucial.

## Introduction

Peritoneal dialysis (PD) is a form of renal replacement therapy for patients with end-stage renal disease (ESRD). This method may be chosen over hemodialysis as it provides lower costs, increased early treatment survival, and better patient autonomy and flexibility for activities of daily life. While proven effective in managing ESRD, PD carries an increased risk of infection, most commonly as peritonitis, often secondary to poor technique [1]. Clinical manifestations of peritonitis typically include cloudy peritoneal effluent, abdominal pain, and fever. Interestingly, a small subset of patients may present with “silent” peritonitis, or peritonitis in the absence of fever and abdominal pain, which can significantly complicate early diagnosis and treatment [2]. 

Identification of a causative organism can be an added barrier further delaying care to patients with PD peritonitis. Commonly, PD peritonitis is caused by gram-positive organisms such as coagulase-negative Staphylococcus and Streptococcus species. These bacteria are predominant in the normal flora of human skin and impose a high risk for PD patients due to their ability to create biofilms on nonbiologic surfaces [3]. 

A rare cause of peritonitis, however, beta-hemolytic streptococci group G is a bacteria present on the skin and in the urogenital and gastrointestinal tracts. Previously thought to be nonpathogenic, group G streptococci have been a topic of interest as instances of life-threatening infections by this pathogen have increased in recent years. Still, few studies have explored the relationship between this organism and its effects on the presentation of PD peritonitis [4]. 

It is important to remain cognizant of PD peritonitis in patients receiving PD therapy even in the absence of classic symptomatology. Advanced age as well as other comorbidities, such as dementia, altered mentation from sepsis, or uncommon organisms may create an atypical presentation, thereby delaying diagnosis and making early detection and treatment difficult. In this report, we explore a case of beta-hemolytic group G streptococci PD-associated peritonitis in a patient with concomitant group G Streptococcus bacteremia. 

## Case presentation

A 95-year-old female presented to our institution’s emergency department with a two-day history of worsening dyspnea and cough, with associated lower leg pain and swelling. She had been discharged from a rehabilitation facility 48 hours prior, after an extensive stay following a recent stroke. Due to the patient’s altered mentation, most of the history was obtained through her next of kin and through chart review. They reported the patient appeared more fatigued compared to baseline, with increasing confusion and cough. They were also concerned about a draining left leg wound sustained at the rehabilitation facility. 

**Figure 1:**
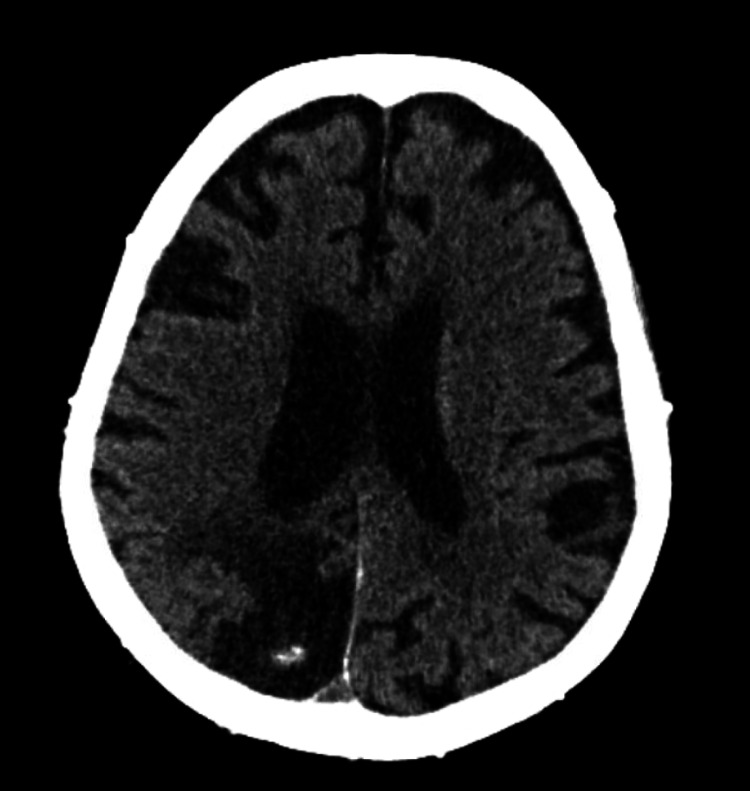
CT head without contrast

Her past medical history was significant for hypertension, hyperlipidemia, hypothyroidism, coronary artery disease (CAD) with previous coronary artery bypass grafting (CABG), congestive heart failure (CHF), aortic valve disease with transcutaneous aortic valve replacement (TAVR), type 2 diabetes mellitus, ESRD on peritoneal dialysis (PD), and a recent stroke with lasting speech and right-sided motor deficits. A CT scan of her head showed stable chronic infarct changes and volume loss (Figure 1). Her social history was negative for alcohol or tobacco use. Code status was confirmed with the patient's power of attorney (POA), and the patient remained “full code.” The review of systems was positive for dyspnea, wheezing, chest pain, and swelling of the lower extremities and negative for fever, cough, nausea, vomiting, or abdominal pain. 

The patient was hypertensive on admission, mildly febrile at a maximum temperature of 100 F (37.8 C), with an oxygen saturation of 94% on a 2L nasal cannula. On physical exam, the patient appeared in moderate distress. She was oriented to name and place only. A lung exam revealed diffuse wheezing and crackles. The cardiac exam showed a regular rate and rhythm with a normal S1 and S2 and no audible murmurs. Her lower extremities showed 2+ pitting edema bilaterally. A shallow, ulcerated wound was found on the left lateral leg just distal to the knee. The lesion was warm to the touch with purulent drainage and a small amount of erythema. Her abdomen was soft, nontender, and nondistended with a PD catheter in place. A neurological exam showed garbled speech with appropriate responses to commands. 

**Figure 2:**
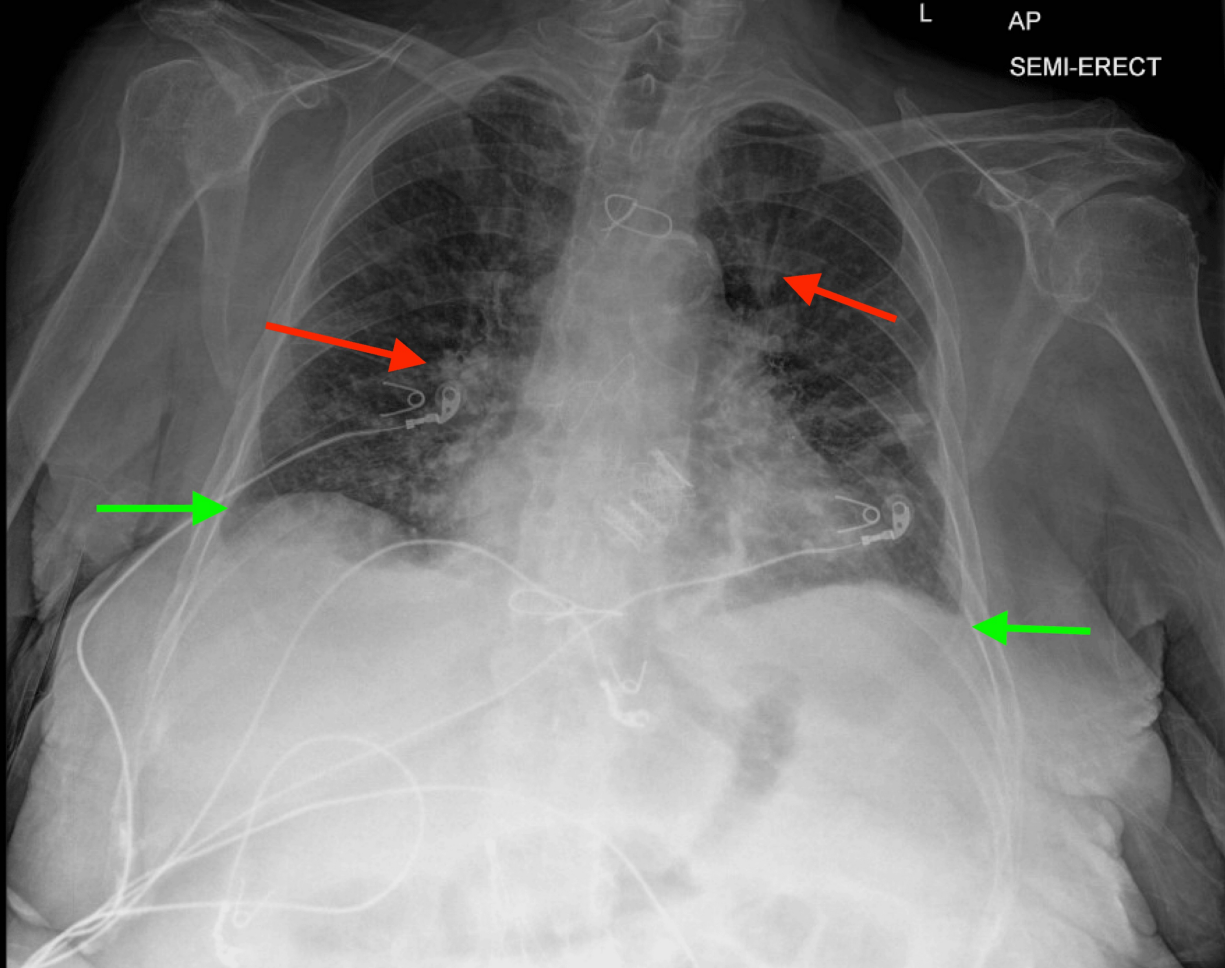
Initial chest X-ray imaging red arrows: interstitial prominence; green arrows: small pleural effusions

Initial imaging included a chest X-ray, which showed mild interstitial prominence suggestive of pulmonary edema and small bilateral pleural effusions, as seen in Figure 2. Ultrasound of the lower extremity wound showed no concern for abscess. Initial laboratory testing was significant for leukocytosis with left shift (17,000/uL with 9% bands) and anemia (hemoglobin of 10.7 g/dL). The platelet count was normal (172 × 109/L). Basic chemistry was consistent with a patient on peritoneal dialysis. Nasal PCR was positive for respiratory syncytial virus (RSV).

In addition to supportive care for her RSV and continued management of her chronic conditions, the patient was started on empiric intravenous vancomycin for her lower extremity wound. Yet, her leukocytosis worsened to 20,000/uL with 13% bands, and blood cultures grew gram-positive cocci in chains. Cefepime was added. Preliminary wound smears showed gram-positive cocci as well, and wound PCR was negative for methicillin-resistant Staphylococcus aureus (MRSA).

On the third hospital day, beta-hemolytic group G Streptococcus was grown on blood culture, and intravenous cefepime was exchanged for intravenous ceftriaxone. Following the lack of significant improvement of leukocytosis, investigation of other potential sources of infection led to peritoneal dialysate fluid analysis, which showed 401 WBCs/uL with 60% segmented neutrophils suspicious for peritonitis. No documentation indicated any change in the appearance of the dialysate; however, a delay in the exchange process was noted due to difficulty setting up the equipment. The patient remained afebrile, with a persistently elevated WBC count (18,000 u/L), stable hemodynamics, and no clinical signs of peritonitis. Intraperitoneal antibiotics were initiated with 1 g of ceftriaxone added to the patient’s nightly PD dwell. Prophylactic fluconazole 100 mg daily was also initiated. Peritoneal dialysate fluid cultures were collected following the administration of antibiotics. 

**Figure 3:**
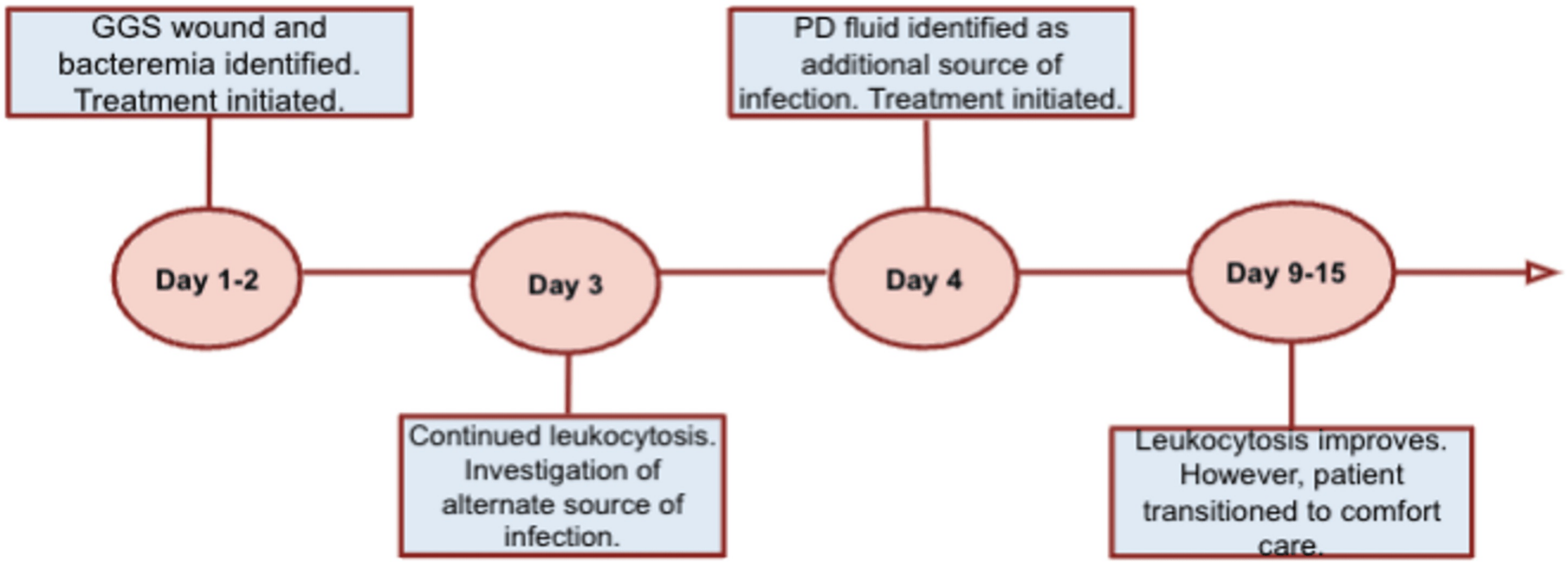
Case timeline GGS: group G Streptococcus; PD: peritoneal dialysis

In the following days, the patient’s leukocytosis gradually resolved. However, she remained encephalopathic and continued to need 2-4 L of oxygen supplementation via nasal cannula. Follow-up blood cultures returned no growth. The repeat peritoneal fluid analysis showed improvement with 30 nucleated cells/uL, and both the initial and repeat peritoneal dialysate fluid cultures returned with no growth to date. Despite these improvements, the patient experienced an acute worsening of respiratory and mental status, requiring intubation and ICU admission. Ultimately, her family decided against escalation of care, and she was transitioned to comfort measures only before passing away shortly thereafter. The timeline of this patient's presentation is demonstrated in Figure 3.

## Discussion

The case of this 95-year-old female describes a challenging and not uncommon scenario. Our patient presented with dyspnea and a lower extremity wound and was found to have RSV, bacteremia, and peritonitis. This case was reviewed as a part of the institutional morbidity and mortality process by both the Division of Hospital Medicine and Critical Care. 

It is important to underscore the fragility of this patient’s health prior to this hospitalization, as evidenced by her extensive comorbidities and advanced age. Her altered mental status, likely related to her recent stroke, and encephalopathy further complicated our assessment of the patient. These factors, in the context of a patient who had been recently transferred between several institutions, made her risk for complications very high. For this reason, multiple conversations with the patient and her family were conducted discussing goals of care throughout her hospital course. Ultimately, the decision to transfer her to comfort measures was made only after ICU admission and multiple invasive interventions, highlighting the need for earlier end-of-life discussion in our elderly population. 

The presence of beta-hemolytic group G Streptococcus (GGS) in both the initial wound and blood culture was unexpected, acting as another factor complicating this case. Despite the incidence of group G being lower than group A and group B streptococci, higher mortality rates between 5-30% have been reported [5]. Group G bacteremia is most commonly associated with malignancy and alcoholism. Studies have also seen associations with renal failure, hypertension, diabetes, and liver failure, with skin infections being the most frequent source of GGS bacteremia [6]. Diagnosis is through culture of suspected sources of infection such as blood, sputum, or pleural fluid. Treatment of GGS is with antibiotics, most commonly penicillins, vancomycin, ampicillin, and cefotaxime [7,8]. Because this was a rare pathogen with multiple possible sources of infection, ceftriaxone was used in our patient to provide broader coverage. 

A rare cause of peritonitis, GGS has been proposed to spread hematogenously into the peritoneal cavity [4,9]. At the time of diagnosis, this patient was afebrile and without any clinical signs of peritonitis. It is important to acknowledge the “silent” nature of this peritonitis in the setting of a patient with bacteremia. "Silent" peritonitis is defined as peritonitis in the absence of fever or abdominal pain and can present a significant barrier to the early detection of the condition. Despite being considered an atypical presentation, some literature suggests upwards of 20% of PD-associated peritonitis cases lack these classic features [2,9]. Given this relatively high reported incidence, clinicians must maintain a high index of suspicion for peritonitis in patients undergoing peritoneal dialysis. This is especially important in cases such as this, where other factors such as advanced age and altered mental status make detection more difficult [10]. Importantly, this patient was already being treated with broad-spectrum antibiotics at the time of fluid analysis and culture, likely further masking clinical signs and contributing to negative cultures. 

While a lack of fever and abdominal pain may not reliably rule out peritonitis, other markers, such as cloudy dialysate fluid and cell counts ≥100 cells/μL with ≥ 50% granulocytes, are often present and remain important tools in its diagnosis [11]. Interestingly, it has been reported that “silent” PD-associated peritonitis is more likely to be culture-negative and caused by more mild gram-positive organisms such as staphylococci or streptococci species [2,12]. The “silent” presentation of our patient compared to typical markers of peritonitis is compared in Table 1. This, in addition to obtaining cultures only after antibiotic administration, may explain the lack of GGS growth on PD cultures. Despite these associations, "silent" presentation has not been associated with improved outcomes, with rates of treatment failure and hospitalization similar to non-silent groups [2,12].

**Table 1:** Diagnostic criteria of peritonitis compared to patient presentation PD: peritoneal dialysis; *dialysate cultures collected following antibiotic administration

Diagnostic Criteria [11]	PD Peritonitis	Silent Peritonitis	Our Patient
Clinical features consistent with peritonitis (i.e. fever, abdominal pain, tenderness)	x	−	−
Positive dialysis effluent culture	x	x	−*
Dialysis effluent white cell count > 100/μL or > 0.1 ×× 109 /L with >50% polymorphonuclear leukocytes	x	x	x

Lastly, to the best of our knowledge, there are no reported cases of PD-associated GGS peritonitis. It is unclear if this unusual organism contributed to the atypical presentation described here and whether other cases of GGS peritonitis might present similarly. As infections with GGS continue to rise, more insight into the presentation, diagnosis, and management of this condition will give way to improved patient outcomes.

## Conclusions

Given the significant potential for a “silent” presentation of PD peritonitis, it is important to keep a high index of suspicion in patients undergoing peritoneal dialysis. This is especially true when other factors, such as advanced age and altered mental status, make detection more difficult. There should be a relatively low threshold for dialysate fluid analysis in situations with clinical uncertainty. Additionally, although much remains unknown about pathogens such as GGS, it is possible they may contribute to atypical presentations, as in this case. More research on the topic is required to further elucidate this connection. 

This case also highlights the need for early discussions about end-of-life care in our elderly patients while they have the capacity to be informed decision-makers. When appropriate, the enlistment of our palliative care colleagues can help guide these complex discussions and prevent undue prolongation of suffering.
